# Pregnant women exhibit decreased trigeminal sensitivity

**DOI:** 10.1002/brb3.3597

**Published:** 2024-07-02

**Authors:** Agnieszka Sabiniewicz, Michał Pieniak, Thomas Hummel

**Affiliations:** ^1^ Department of Otorhinolaryngology, Smell & Taste Clinic TU Dresden Dresden Germany; ^2^ Department of Historical and Pedagogical Sciences, Institute of Psychology University of Wroclaw Wroclaw Poland

**Keywords:** olfaction, pregnancy, trigeminal

## Abstract

**Introduction:**

Chemosensory function in pregnant women is far from being fully understood due to the lack of data and inconsistencies between the results of self‐reports and objective studies.

**Methods:**

In the present study in pregnant and non‐pregnant women (*n*_pregnant_ = 14, *n*_non‐pregnant_ = 13), we measured EEG‐derived electrophysiological response measures supported by psychophysical olfactory and trigeminal tests.

**Results:**

Results indicate that the olfactory event‐related potential amplitudes or latencies of the P1, N1, and P2 components remain unchanged in pregnant women. In accordance with these findings, no difference was observed between pregnant and non‐pregnant women in psychophysical olfactory tests. However, pregnant women displayed a lower degree of sensitivity to trigeminal stimuli compared to non‐pregnant controls, which was also reflected in the electrophysiological responses to trigeminal stimuli.

**Conclusion:**

Counterintuitive as they may seem, our findings demonstrate a “flattening” of chemosomatosensory responses. Psychological processes occurring during pregnancy, such as changes in socioemotional perception of odors resulting from the diminished stress response, may provide a background to these results. Overall, the present results indicate the absence of major differences between non‐pregnant and pregnant women in terms of measured olfactory function though chemosomatosensory function of the pregnant women appears to be decreased.

## Graphical abstract

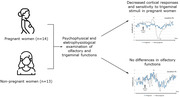

## 1 INTRODUCTION

Pregnant women frequently report abnormalities in their olfactory functions (Kölble et al., 2001; Laska et al., 1996; Pletsch & Thornton Kratz, 2004). More precisely, questionnaire‐based studies demonstrate that about two‐thirds of pregnant women declare alterations in their smell perception, overall increased smell sensitivity (Cameron, 2007; Nordin et al., 2004), and specific sensitivity to one or more odors (Nordin et al., 2007). As early as 1930, in one of the first scientific studies in the field, Henssge (1930) reported a case of a 27‐year‐old pregnant woman who declared that her olfactory “sensitivity increased” and that odors that were “normally imperceptible were now unbearable” (Cameron, 2014).

Self‐ratings of the sense of smell do not reflect actual olfactory abilities, as measured with psychophysical olfactory tests (Landis et al., 2003; Lötsch & Hummel, 2019). Accordingly, to date, no studies among the few conducted with standardized and validated olfactory tests like the Sniffin’ Sticks (Hummel et al., 1997; Oleszkiewicz et al., 2019) or the University of Pennsylvania Smell Identification Test (UPSIT; Doty et al., 1984) have demonstrated an enhancement in olfactory function in pregnancy, while some indicated olfactory impairment or hyposmia (Ochsenbein‐Kölble et al., 2007; Savovic et al., 2002; Şimşek et al., 2021; Tan et al., 2020; Yasar et al., 2016). On the other hand, several studies reported no differences in any olfactory measure between pregnant and non‐pregnant women (Cameron, 2007, 2014; Fornazieri et al., 2019; Gilbert & Wysocki, 1991; Kölble et al., 2001; Laska et al., 1996). Recently, Albaugh et al. (2022), in a systematic meta‐analysis including data from different trimesters in pregnancy and diverse populations, found that the only difference between pregnant and non‐pregnant women was impaired odor identification in case of the pregnant women. This finding was also confirmed by the newest meta‐analysis in this field, conducted by Muluh et al. (2024).

Considering that the sense of smell is vital for protecting us from environmental hazards and risky ingestive behaviors (Stevenson, 2010), supposedly protecting the fetus at the early stage (Profet, 1992), the results of the aforementioned studies seem counterintuitive. One may expect pregnant women to display increased sensitivity toward the chemical environment, but this does not manifest itself on the level of olfactory functions. At the same time, chemosomatosensation mediated by the trigeminal nerve plays a major role in sensitivity to the chemical environment (Hummel & Livermore, 2002). Despite this, its functions in pregnant women are far from being understood. To date, only Olofsson et al. (2005) have investigated the nasal chemosensory trigeminal system in pregnant women. Contrary to their expectations, EEG‐derived chemosensory event‐related potentials (ERP) did not display any differences in trigeminal function between pregnant and non‐pregnant women (Olofsson et al., 2005). However, it should be mentioned that Olofsson and colleagues used pyridine as a trigeminal stimulus, which, in fact, is a mixed olfactory/trigeminal stimulus. Thus, each response was confounded by olfactory activation, and a clear division between olfactory and trigeminal responses was not possible.

Hence, the aim of the present study was to extensively investigate chemoperception. We examined olfactory and trigeminal functions separately, that is, using selective olfactory and trigeminal stimuli in pregnant versus non‐pregnant women, via electrophysiological response supported by psychophysical tests. We expected that such a combination of measures of reactions toward olfactory and trigeminal stimuli would allow a more profound understanding of chemosensory response in pregnant versus non‐pregnant women. More specifically, we hypothesized that pregnant versus non‐pregnant women would not differ in terms of olfactory responses but that there would be differences in terms of trigeminal activation.

## 2 MATERIALS AND METHODS

### 2.1 Participants

Fourteen pregnant and 13 non‐pregnant women aged 23–37 years were enrolled to participate in the study. The sample size was determined by utilizing G*power software (Faul et al., 2007). In one‐way analysis of variance for two groups, with a level of significance set to *α* = .05 (described in detail in 2.5 Statistical analyses) to detect large effects of *f* = 0.9 (critical *F* = 4.4), the projected sample size was at least 20.

Inclusion criteria for eligible subjects were age ≥18 years, comprehension of the benefits and risks of participation in the study, good general health, and normal olfactory function prior to pregnancy. Exclusion criteria would be severe health problems that would affect the sense of smell, for example, acute or chronic rhinosinusitis, neurodegenerative disease like Parkinson's disease or Alzheimer's disease, myasthenia gravis, epilepsy, uncontrolled diabetes mellitus, and major renal dysfunction. All participants were tested in accordance with the Declaration of Helsinki on Biomedical Studies Involving Human Subjects. Informed consent was obtained from all participants prior to their inclusion in the study. The research protocol has been approved by the Ethics Review Board at the Medical Faculty of the TU Dresden [EK11042013].

Because of excessive artifacts in the collected EEG signal, data from some participants could not be analyzed. The final dataset comprised 23 recordings of olfactory ERPs (11 pregnant and 12 non‐pregnant) and 21 recordings of trigeminal ERPs (10 pregnant and 11 non‐pregnant). Pregnant women were in their second trimester of the pregnancy (gestational age: range 13–27 weeks, *M *= 21.5 ± 5.52 weeks) and were slightly older than non‐pregnant women (*M*_pregnant _= 30.5 ± 4.6 years, *M*_non‐pregnant _= 25.6 ± 2.5 years; *t*[19] = 3.12, *p *= .006). None of the participants had olfactory dysfunction as indicated by psychophysical olfactory test results (diagnostic criteria described below).

### 2.2 ERP acquisition

#### 2.2.1 Stimulus presentation

For selective olfactory stimulation, we used phenylethyl alcohol (PEA; 40%, v/v), and for selective trigeminal stimulation, we used carbon dioxide (CO_2_, 50%, *v*/*v*) (Fröhlich, 1851; Kobal & Hummel, 1988). Both stimuli were delivered by a computer‐controlled olfactometer (OM6b, Burghart MT) at a flow of 6 L/min. They were embedded into a constant stream of warmed (36°C) and humidified (80% relative humidity) air. Air was delivered to one of the nostrils through Teflon tubing (3 mm inner diameter). Stimulus duration was 250 ms for PEA and 200 ms for CO_2_. Stimuli were delivered at an interstimulus interval of approximately 15–20 s in randomized order, with the final number of 30 trials per stimulus.

Recordings took place in a well‐ventilated room with participants comfortably seated. Participants performed a simple visual tracking task to limit eye blinks and head movements and to maintain vigilance (Kobal et al., 1990). To mask the sounds of the olfactometer, which could suggest upcoming stimulus presentation, participants wore headphones delivering white noise (∼60 dB SPL).

#### 2.2.2 EEG recording and preprocessing

EEG was recorded at positions Fz, Cz, Pz, C3, and C4 of the 10/20 system referenced against earlobes (A1 + A2). Eye blinks were monitored from the Fp2 lead. Participant's skin was cleansed with “Skin Pure” prepping cream (Nihon Kohden) and Ag/AgCl disc electrodes (BioSemi) were attached using self‐adhesive cream (EC2 Grass Electrode Cream; Grass). Each recording started 500 ms before stimulus onset and continued for 1500 ms after the stimulation.

For recordings (8‐channel amplifier; Schabert), a bandpass of 0.2–30 Hz was used. The 2 s stimulus‐linked signals (including a 500 ms pre‐trigger period for determination of baseline) were recorded at a sampling frequency of 512 Hz.

EEG signal preprocessing was conducted with EPEvaluate 4.2.2 (Kobal). The signal was filtered offline (low pass 15 Hz) and visually inspected for motor and eye‐blinking artifacts. For the participants included in the analyses, the number of discarded trials ranged between 6 and 25 (*M* = 14.6, SD = 5.7) for the olfactory stimulation and between 3 and 23 (*M* = 15.5, SD = 5.8) for the trigeminal stimulation. P1, N1, and P2 peaks were identified by trained observers (M. P. and T. H.). Stopband attenuation was set to 50 Hz. For each site, both peak latencies, base‐to‐peak amplitudes for P1, N1, and P2 components as well as peak‐to‐peak P1‐N1 and N1‐P2 amplitudes, were measured. P1 and N1 components represent cortical activity related to the quality of the stimulus, whereas P2 represents an interpretation of the novelty and significance of the stimulus (Rombaux et al., 2006).

### 2.3 Psychophysical evaluation

#### 2.3.1 Olfactory functions

Olfactory functions were assessed with Sniffin’ Sticks battery, which comprises three tests measuring distinct aspects of olfactory perception—olfactory threshold, odor discrimination, and odor identification (Hummel et al., 1997; Oleszkiewicz et al., 2019). All odors were presented in pen‐like odor dispensers.

The threshold test measures sensitivity to PEA, a relatively selective olfactory stimulant. Participants are presented with three pens in randomized order. One of the three pens contains PEA, and two contain the solvent (odorless propylene glycol). The task is to indicate which pen contains the target odor. PEA is presented in 16 dilutions starting from 4% PEA dilution in propylene glycol and diluted further in 1:2 ratio. A reverse staircase procedure with seven turning points is employed, and the threshold score (*T*) ranging from 1 to 16 is obtained as an average of the last four reversals (higher scores indicate greater sensitivity to PEA).

The discrimination test involves 16 trials in which the participant is presented with triplets of pens containing suprathreshold odors—two pens with the same odorant and one pen with a distinct odorant. The task is to indicate which pen differs from the other two. Each correct answer is scored with 1 point, and the total discrimination score (D) ranges from 0 to 16.

In the identification test, the participant identifies an odor presented at a suprathreshold concentration from a set of four visual and labeled cues (one correct and three incorrect). Sixteen different odors are presented, and each correct answer is scored with 1 point. The total identification score (I) ranges from 0 to 16.

Scores in threshold, discrimination, and identification tests are further summed up to an overall total olfactory function (TDI) score ranging from 1 to 48. In women aged 21–40 years, a score of 31 points is treated as a cutoff score for hyposmia (Oleszkiewicz et al., 2019).

#### 2.3.2 Trigeminal functions

Trigeminal functions were assessed with a “lateralization test” (Hummel et al., 2003). In this task, both nostrils are stimulated simultaneously—one nostril with non‐diluted eucalyptol, which stimulates the trigeminal nerve, and the other nostril with odorless propylene glycol. The stimulated side is assigned randomly. The stimuli are presented in two polyethylene squeeze bottles (250 mL volume) with a spout. Both spouts are placed in the nostrils during each trial, and the bottles are simultaneously squeezed with a hand‐held squeezing device. The participant's task is to determine which nostril received an odorant. This test consists of 20 trials, and for each correct answer, the participant scores 1 point.

Additionally, participants rated the CO_2_ stimulation used during olfactometry to evoke trigeminal ERP in terms of stimulus intensity, pleasantness, and painfulness on scales from 0 to 10.

### 2.4 Questionnaire testing

#### 2.4.1 Individual significance of olfaction

We used individual significance of olfaction questionnaire (ISOQ) characterized by good internal reliability (Cronbach's *α *= .77) to assess the individual importance of the sense of smell (Croy et al., 2010). The ISOQ is composed of 18 items grouped into three scales—“Association,” “Application,” and “Consequence.” The Association scale measures whether a person is susceptible to having their emotions and memories triggered by odors. The Application scale assesses the degree to which a person uses their sense of smell in everyday tasks. The Consequence scale refers to how the odors influence a person's daily decisions and impressions. Participants used 4‐point Likert‐type scale ranging from 1 (“I totally disagree”) to 4 (“I totally agree”), and the responses within each scale were summed.

#### 2.4.2 Depressive symptoms

Depressive symptoms were measured with Beck Depression Inventory II (BDI‐II)—a 21‐item questionnaire with good internal reliability (Cronbach's *α *= .92) (Beck et al., 1996). In BDI‐II, participants rate the severity of different symptoms of depression occurring within 2 weeks prior to testing on Likert‐type scale ranging from 0 to 3. Responses were averaged and ranged from 0 to 3. Depressive symptoms were assessed as a control variable based on reports demonstrating olfactory changes accompanying depression (Croy & Hummel, 2017; Kohli et al., 2016; Sabiniewicz et al., 2022).

### 2.5 Statistical analyses

All statistical analyses were conducted with jamovi 2.2.5 for Windows with a level of significance set to *α *= .05. To compare pregnant and non‐pregnant women's scores in psychophysical tests and questionnaires, we used Student's *t*‐test for independent samples. To evaluate between‐group differences in chemosensory ERPs, we ran a series of mixed‐model analysis of variances with pregnancy as a between‐subject factor and electrode location as a within‐subject factor. Separate models have been run for P1, N1, and P2 base‐to‐peak amplitudes and latencies, and P1‐N1, N1‐P2 peak‐to‐peak amplitudes for both PEA and CO_2_. All post hoc analyses were Bonferroni corrected for multiple comparisons. Full model coefficients for all the analysis of variance models are presented in Supporting Information File S1. Finally, to measure the relationship between scores in psychophysical tests and chemosensory ERPs, we ran a series of Pearson's correlation analyses, separately for TDI scores and olfactory ERPs and for lateralization scores and trigeminal ERPs.

## 3 RESULTS

Descriptive statistics describing scores in the psychophysical olfactory and trigeminal tests, as well as in psychological questionnaires, are presented in Table 1.

**TABLE 1:** Descriptive statistics of the psychophysical and psychological measures.

	Pregnant			Non‐pregnant			*t*‐test *p* value
	M	SD	*n*	M	SD	*n*	
Age (in years)	30.5	4.6	10	26.3	3.4	12	.021*
Olfactory threshold	9	1.6	11	8.3	2.2	13	.353
Odor discrimination	14.5	1.2	11	13.8	1.3	13	.261
Odor identification	14.5	1.1	11	14.2	1.5	13	.693
TDI	37.9	1.8	11	35.9	3.2	13	.076
ISOQ association	16.7	3.1	10	18.3	3.6	10	.298
ISOQ application	16.1	1.8	10	17.7	1.8	10	.060
ISOQ consequence	17.1	1	10	16.4	2.5	10	.422
BDI‐2	1.1	1.4	11	.8	.9	13	.606
Lateralization	11.6	2.9	10	15	3.3	13	.018*
CO_2_ intensity	2.9	2.9	11	5.7	3.2	13	.037*
CO_2_ pleasantness	2.5	2.3	11	3.3	2	13	.347
CO_2_ pain	1.8	2.6	11	4.6	3.8	13	.048*

Abbreviations: BDI, beck depression inventory; ISOQ, individual significance of olfaction questionnaire. Asterisk stands for p < .05.

### 3.1 Psychophysical evaluation

A series of Student's *t*‐tests for independent samples showed that pregnant and non‐pregnant women did not differ significantly in terms of olfactory test results (for olfactory threshold: *t*[22] = .95, *p *= .35, Cohen's *d *= .39; for odor discrimination: *t*[22] = 1.15, *p *= .26, Cohen's *d *= .47; for odor identification: *t*[22] = .40, *p *= .69, Cohen's *d *= .16; for overall TDI score: *t*[22] = 1.86, *p *= .076, Cohen's *d *= .76).

However, there was a statistically significant difference in trigeminal sensitivity obtained with the lateralization test (*t*[21] = 2.57, *p *= .018, Cohen's *d *= 1.08) with pregnant women showing less sensitivity (*M *= 11.6 ± 2.88) than non‐pregnant women (*M *= 15 ± 3.34). Additionally, we found that non‐pregnant women rated the CO_2_ stimulation as more intense (*t*[22] = 2.22, *p *= .037, Cohen's *d *= .91; *M*_non‐pregnant _= 5.69 ± 3.17; *M*_pregnant _= 2.91 ± 2.91) and more painful (*t*[22] = 2.09, *p *= .048, *d *= .86; *M*_non‐pregnant _= 4.62 ± 3.75; *M*_pregnant _= 1.82 ± 2.56) than pregnant women; however, there was no significant group difference in the ratings of the CO_2_ stimulus pleasantness (*t*[22] = .96, *p *= .35, *d *= .39).

### 3.2 Questionnaire testing

A series of Student's *t*‐tests for independent samples demonstrated that pregnant and non‐pregnant women did not differ significantly in terms of either ISOQ questionnaire's scales (for “Association”: *t*[18] = 1.07, *p *= .298, Cohen's *d *= .48, for Application: *t*[18] = 2.01, *p *= .06, Cohen's *d *= .90; for “Consequence”: *t*[18] = .82, *p *= .422, Cohen's *d *= .37) scales. Additionally, no differences have been found in the severity of depressive symptoms (*t*[22] = .52, *p *= .606, Cohen's *d *= .21).

### 3.3 Olfactory ERPs

**FIGURE 1:**
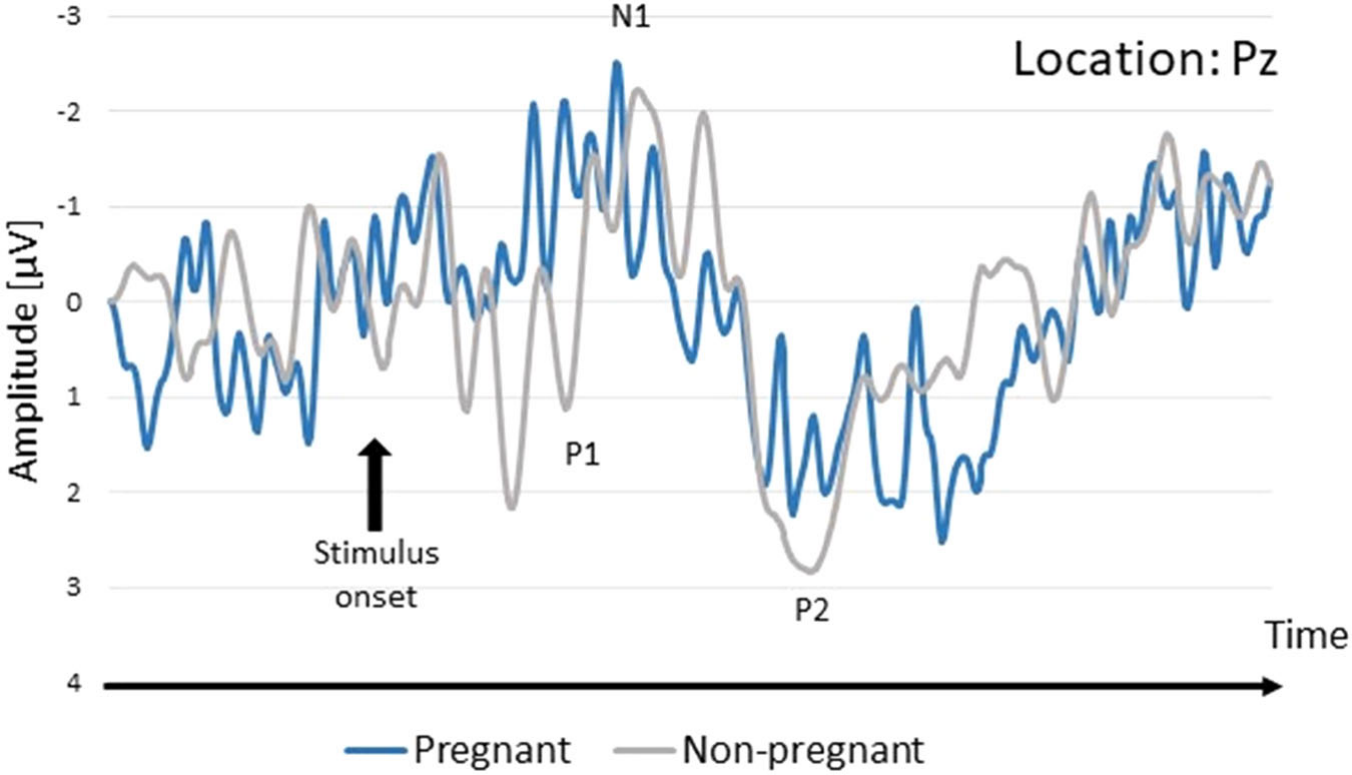
Schematic representations of the olfactory event‐related potentials across the study groups recorded at Pz.

None of the mixed‐model analysis of variance showed a significant main effect of pregnancy or significant effect of pregnancy X location interaction for P1, N1, P2 base‐to‐peak amplitudes, latencies, or P1‐N1 and N1‐P2 peak‐to‐peak amplitudes (all *p*s > .05; full model coefficients and figures presenting all ERPs components are reported in Supporting Information File S1). The grand‐average ERPs are presented in Figure 1.

### 3.4 Trigeminal ERPs

**FIGURE 2:**
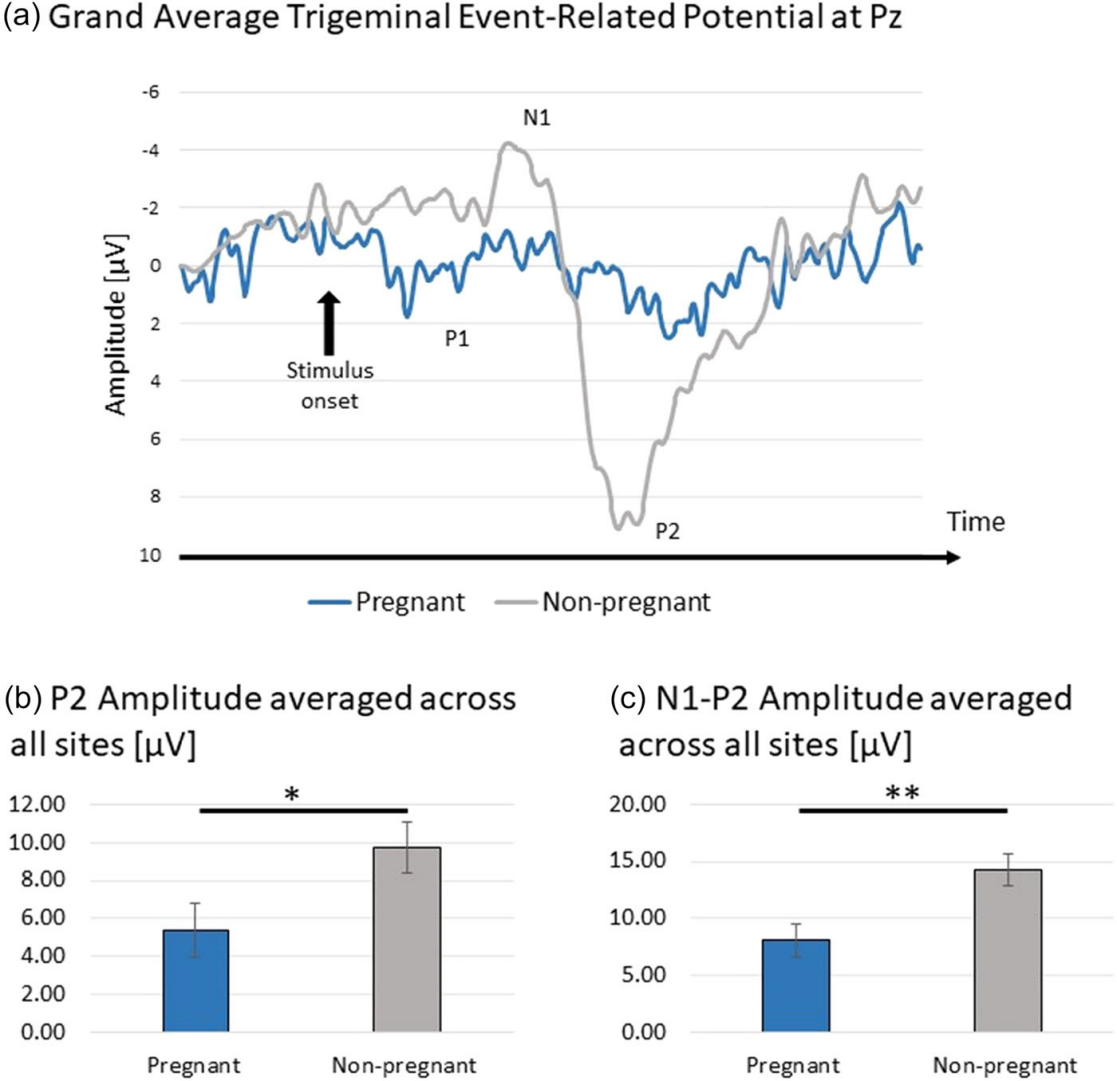
Schematic representations of the between‐group differences in trigeminal event‐related potentials recorded at Pz (panel a), base‐to‐peak P2 amplitude averaged across all recording sites (panel b), and N1‐P2 peak‐to‐peak amplitude averaged across all recording sites (panel c). *−*p *< .05; **−*p *< .01.

We observed a significant main effect of pregnancy on base‐to‐peak P2 amplitude (*F*[1, 19] = 5.16, *p *= .035, *η* ^2^_p _= .21). Post hoc analysis showed that the amplitude was greater in the non‐pregnant group (*M *= 9.75 ± 1.33) than in the pregnant group (*M *= 5.37 ± 1.40; *p *= .035, Bonferroni corrected). Additionally, we found an analogous significant effect of pregnancy on N1‐P2 peak‐to‐peak amplitude (*F*[1, 19] = 9.17, *p *= .007, *η* ^2^_p _= .33). Post hoc analysis demonstrated that the amplitude was greater in the non‐pregnant group (*M *= 14.18 ± 1.39) than in the pregnant group (*M *= 8.07 ± 1.46; *p *= .007, Bonferroni corrected). These results are presented in Figure 2. There was no significant effect of pregnancy or interaction effect on any of the peak latencies (all *p*s > .05; full model coefficients and figures presenting all ERPs components are reported in Supporting Information File S1).

### 3.5 Correlation analyses

We found no significant correlations between odor discrimination and identification and any of the olfactory ERP components (all *p*s > .05). However, the analyses systematically revealed a significant moderate negative correlation between scores in the threshold test and latencies of N1 and P2 components. This effect was observed for all recording positions (except P2 latency at Fz); the higher the olfactory sensitivity, the shorter the latency for these two ERPs components. Pearson's *r* coefficients ranged from −.48 (*p *= .021) to −.42 (*p *= .045). We did not find any systematic relationship between olfactory sensitivity measured with other psychophysical tests and the amplitude of any of the ERP components.

For the trigeminal perception, we systematically found a significant moderate positive relationship between scores in the lateralization test and both P1‐N1 and N1‐P2 peak‐to‐peak amplitudes. This effect was observed for all electrode locations; the better the lateralization abilities, the larger the peak‐to‐peak amplitude. Pearson's *r* coefficients ranged from.43 (*p *= .045) to.64 (*p *= .002). Additionally, we found a significant moderate positive correlation between intensity ratings of the CO_2_ stimulus employed in EEG recordings and N1‐P2 peak‐to‐peak amplitude for all the electrode locations except Fz (Pearson's *r* coefficients ranged from.47 [*p *= .028] to.49 [*p *= .018]). We did not find any systematic relationship between trigeminal perception measured with psychophysical methods and the latency of any of the ERP components.

## 4 DISCUSSION

Chemosensory function in pregnant women requires a more profound understanding in the context of the inconsistent results of self‐reports and objective studies. Here, we first investigated an assumption that pregnant versus non‐pregnant women would not differ in terms of olfactory response. Second, we decided to explore the role of trigeminal response via a selective trigeminal stimulant. Our approach in both cases included electrophysiological response measures supported by a series of psychophysical tests. In addition, we measured the relationship between scores in psychophysical tests and chemosensory ERP, separately for TDI scores and olfactory ERP and for lateralization scores and trigeminal ERP. Regarding the first hypothesis, the present data support the view that the olfactory ERP amplitudes or latencies of the N1, P2, and P2 components remain unchanged in pregnant women. In line with these findings, pregnant and non‐pregnant women performed equally well in psychophysical olfactory tests.

The results of the present study are in accord with most of the literature on women's sense of smell during pregnancy. Despite anecdotal evidence and self‐reported data, studies based on objective olfactory assessment do not confirm changes in olfactory sensitivity in pregnancy (Albaugh et al., 2022; Cameron, 2007, 2014; Fornazieri et al., 2019; Gilbert & Wysocki, 1991; Kölble et al., 2001; Laska et al., 1996). Here, we strengthen these findings by demonstrating no differences in electrophysiological response to olfactory stimuli in pregnant versus non‐pregnant women. This implies that early sensory processes do not contribute to abnormal chemosensory perception in pregnant women (Olofsson et al., 2005). Instead, we suggest that alterations in cognitive functioning might be the source of anomalous olfactory perception.

Pregnancy involves long‐lasting changes in human brain structures (Hoekzema et al., 2017) that might translate into cognitive alterations (Duarte‐Guterman et al., 2019). For example, meta‐analyses have demonstrated that pregnant women display small but significant deficits in free recall, delayed free recall, working memory, and executive function (Anderson & Rutherford, 2012; Davies et al., 2018). A systemic review of 38 studies conducted by Ouellette and Hampson (2019) confirmed a modest reduction in memory function in pregnant women, that is, verbal recall, working memory, and prospective memory. Furthermore, pregnant women's spatial navigation performance is lower than non‐pregnant controls (Lisofsky et al., 2016). The link between olfactory and cognitive performance has been demonstrated in men and non‐pregnant women (Challakere Ramaswamy & Schofield, 2022; Danthiir et al., 2001; Larsson et al., 2004). Here, we suggest that future studies, including neuroimaging techniques, could shed further light on the issue of possible cognitive changes related to olfactory perception in pregnant women.

Contrary to the findings in the olfactory system, we found a systematic pattern of results indicating decreased sensitivity of the trigeminal system in pregnant women. In the present study, pregnant women scored less in the lateralization test and had lower subjective ratings of intensity and painfulness of a trigeminal stimulus. More importantly, the electrophysiological responses to trigeminal stimuli displayed the same pattern, indicating reduced strength (amplitude) of the electrophysiological response in pregnancy. Importantly, changes in the trigeminal ERP were observed across both the sensory components of the response, as well as components related to the interpretation of significant meaning. Based on the present results, we suggest that while pregnant women do not differ from non‐pregnant ones in terms of olfactory function, their chemosomatosensory function is decreased. Furthermore, the correlation results consistently indicated a decrease in trigeminal sensitivity in pregnant women. The nasal chemosomatosensory system warns us from potentially hazardous chemicals with sensations such as irritation, tickling, burning, warming, cooling, and stinging in the nasal and oral cavity and the cornea. These sensations alert the organism to potentially harmful stimuli (Doty & Cometto‐Muniz, 2003). Pregnant women report hypersensitivity to substances that activate primarily the trigeminal system (Broman et al., 2003; as cited in Olofsson et al. [2005]). Still, so far, the only research conducted in this field has demonstrated no differences in trigeminal functions of pregnant versus non‐pregnant women (Olofsson et al., 2005). On the contrary to these notions, based on psychophysical and electrophysiological tests, we demonstrated that sensitivity to trigeminal stimuli was lower in pregnant women compared to non‐pregnant controls. Counterintuitive as they may seem, our findings demonstrate a “flattening” of chemosomatosensory responses. Although these findings indicate changes in cortical responses toward trigeminal stimulus, we speculate that psychological processes that occur during pregnancy may also provide a background to these results. More specifically, pregnancy was demonstrated to cause changes in socioemotional perception of odors, such as decreased and altered responses to chemosensory anxiety signals (Lübke et al., 2017). We suggest that such diminished stress response emerging during pregnancy (Christian, 2012; de Weerth & Buitelaar, 2005) may translate into decreased, erroneous chemosensory perception, thereby protecting women from stress caused by the perception of potentially harmful stimuli. Another plausible explanation is that the decreased responsiveness of the intranasal trigeminal system demonstrated in the present study might be part of an adaptive process of the body's pain system to prepare for childbirth, as indicated by some preliminary evidence on animals (Olza et al., 2020). The decreased responsiveness of the trigeminal system may partly be due to changes in progesterone levels, which are notably elevated during all stages of pregnancy. In fact, progesterone has been shown to modulate central nervous processing of painful stimuli (Manson, 2010), also through activation of opioid receptors at the level of the brain stem/spinal cord (Dawson‐Basoa & Gintzler, 1993, 1996).

The present study is not free from limitations. Further studies in this field should benefit from including a higher number of participants, particularly in the context of many contradictory findings regarding the sense of smell in pregnant women. Due to the small sample size, the present study aimed to detect only large effects. As we were running multiple *t*‐tests when comparing scores in the psychophysical test, a *p*‐value correction shall be considered. However, with the small sample size obtained in our study, such an approach seemed too conservative. Furthermore, null result would carry more weight if the sample size were clearly large enough that a significant finding could have been observed. Additionally, it should be noted that all the participants were in their second trimester of pregnancy. While it made our sample homogenous, based on the current evidence, we cannot exclude that the investigated effects might vary in different trimesters, and the present findings may not be generalized to early pregnancy. Future studies should include women in various stages of pregnancy and, in addition, control for the menstrual cycle phase of the control group. Finally, in the present study, olfactory and trigeminal ERPs were only analyzed in the time domain, which is limited by the presence of latency jitter (Huart et al., 2012). This issue may be tackled by using time‐frequency analysis; however, such analysis could not be performed with the collected dataset due to software limitations.

In conclusion, this study is one of few conducted to date examining brain ERP in pregnant women and the first to focus on their responses to purely olfactory or purely trigeminal stimuli, allowing to investigate their olfactory and trigeminal functions separately. Taken together, the present results suggest that while pregnant women do not differ from non‐pregnant ones in terms of measured olfactory function, their chemo‐somatosensory function is decreased. These findings can be interpreted in terms of changes of progesterone levels, which have been shown to modulate responses to painful stimuli, but also in the context of potential cognitive changes that occur in pregnancy and, respectively, diminished stress response aimed to protect pregnant women from the perception of potentially harmful stimuli.

## AUTHOR CONTRIBUTIONS

**Agnieszka Sabiniewicz**: Writing—original draft; writing—review and editing. **Michał Pieniak**: Writing—original draft; writing—review and editing; data curation; formal analysis. **Thomas Hummel**: Conceptualization; supervision; writing—original draft; writing—review and editing; data curation; formal analysis.

## CONFLICT OF INTEREST STATEMENT

The authors declare no conflicts of interest.

### PEER REVIEW

The peer review history for this article is available at https://publons.com/publon/10.1002/brb3.3597

## Supporting information

Supporting Information

## Data Availability

The datasets analyzed during the present study are not publicly available due to the privacy of the participants but are available from the corresponding author on reasonable request.
