# Supplementary material for: Pregnant women exhibit decreased trigeminal sensitivity
Source: Brain Behav. 2024 Jul 2;14(7):e3597. doi: 10.1002/brb3.3597 (PMC11219288; doi:10.1002/brb3.3597)

**Supplemnentary File 1**

Table S1. Mixed-model ANOVA coefficients for all the models testing effect of pregnancy on olfactory ERPs.

| ERP Component | Effect | *F* | *df* | *p* | *η*^2^_p_ |
| --- | --- | --- | --- | --- | --- |
| P1 Amplitude | Location | 1.15 | 4, 84 | .340 | .05 |
|  | Pregnancy | .06 | 1, 21 | .808 | <.01 |
|  | Location * Pregnancy | .78 | 4, 48 | .544 | .04 |
| N1 Amplitude | Location | .55 | 4, 84 | .697 | .03 |
|  | Pregnancy | <.01 | 1, 21 | .983 | <.01 |
|  | Location * Pregnancy | .30 | 4, 48 | .879 | .01 |
| P2 Amplitude | Location | 3.22 | 4, 84 | .016 | .13 |
|  | Pregnancy | .02 | 1, 21 | .893 | <.01 |
|  | Location * Pregnancy | .64 | 4, 48 | .633 | .03 |
| P1-N1 Amplitude | Location | 1.70 | 4, 84 | .159 | .08 |
|  | Pregnancy | .06 | 1, 21 | .809 | <.01 |
|  | Location * Pregnancy | .55 | 4, 48 | .698 | .03 |
| N1-P2 Amplitude | Location | 4.85 | 4, 84 | .001 | .19 |
|  | Pregnancy | .01 | 1, 21 | .918 | <.01 |
|  | Location * Pregnancy | 1.06 | 4, 48 | .380 | .05 |
| P1 Latency | Location | 1.26 | 4, 84 | .292 | .06 |
|  | Pregnancy | .57 | 1, 21 | .460 | .03 |
|  | Location * Pregnancy | 1.96 | 4, 48 | .108 | .09 |
| N1 Latency | Location | .37 | 4, 84 | .829 | .02 |
|  | Pregnancy | .11 | 1, 21 | .745 | <.01 |
|  | Location * Pregnancy | 1.24 | 4, 48 | .300 | .06 |
| P2 Latency | Location | .38 | 4, 84 | .822 | .02 |
|  | Pregnancy | .53 | 1, 21 | .476 | .02 |
|  | Location * Pregnancy | .57 | 4, 48 | .683 | .03 |

Table S2. Mixed-model ANOVA coefficients for all the models testing effect of pregnancy on trigeminal ERPs.

| ERP Component | Effect | *F* | *df* | *p* | *η*^2^_p_ |
| --- | --- | --- | --- | --- | --- |
| P1 Amplitude | Location | .95 | 4, 76 | .442 | .05 |
|  | Pregnancy | 3.20 | 1, 19 | .090 | .14 |
|  | Location * Pregnancy | .36 | 4, 76 | .839 | .02 |
| N1 Amplitude | Location | 1.13 | 4, 76 | .349 | .06 |
|  | Pregnancy | 1.40 | 1, 19 | .251 | .07 |
|  | Location * Pregnancy | 1.29 | 4, 76 | .282 | .06 |
| P2 Amplitude | Location | 6.48 | 4, 76 | <.001 | .25 |
|  | Pregnancy | 5.16 | 1, 19 | .035 | .21 |
|  | Location * Pregnancy | 2.16 | 4, 76 | .082 | .10 |
| P1-N1 Amplitude | Location | .31 | 4, 76 | .870 | .02 |
|  | Pregnancy | .01 | 1, 19 | .919 | <.01 |
|  | Location * Pregnancy | 1.35 | 4, 76 | .258 | .07 |
| N1-P2 Amplitude | Location | 5.83 | 4, 76 | <.001 | .24 |
|  | Pregnancy | 9.17 | 1, 19 | .007 | .33 |
|  | Location * Pregnancy | 1.55 | 4, 76 | .198 | .08 |
| P1 Latency | Location | .70 | 4, 76 | .595 | .04 |
|  | Pregnancy | 1.92 | 1, 19 | .182 | .09 |
|  | Location * Pregnancy | 1.60 | 4, 76 | .183 | .08 |
| N1 Latency | Location | .57 | 4, 76 | .688 | .03 |
|  | Pregnancy | 1.13 | 1, 19 | .301 | .06 |
|  | Location * Pregnancy | .39 | 4, 76 | .817 | .02 |
| P2 Latency | Location | 1.56 | 4, 76 | .194 | .08 |
|  | Pregnancy | 1.48 | 1, 19 | .239 | .07 |
|  | Location * Pregnancy | .51 | 4, 76 | .731 | .03 |

Figure S1. Averaged amplitudes and latencies of the olfactory ERPs components. Error bars depict standard deviations.


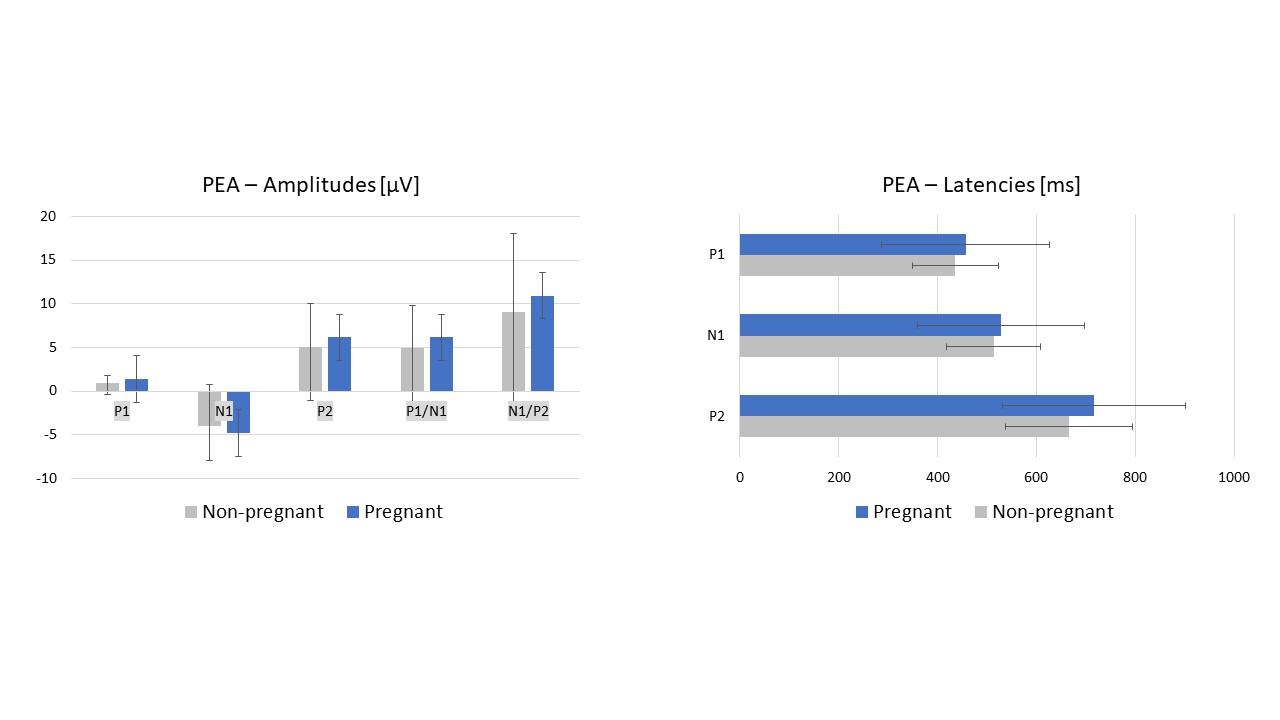


Figure S2. Averaged amplitudes and latencies of the trigeminal ERPs components. Error bars depict standard deviations.


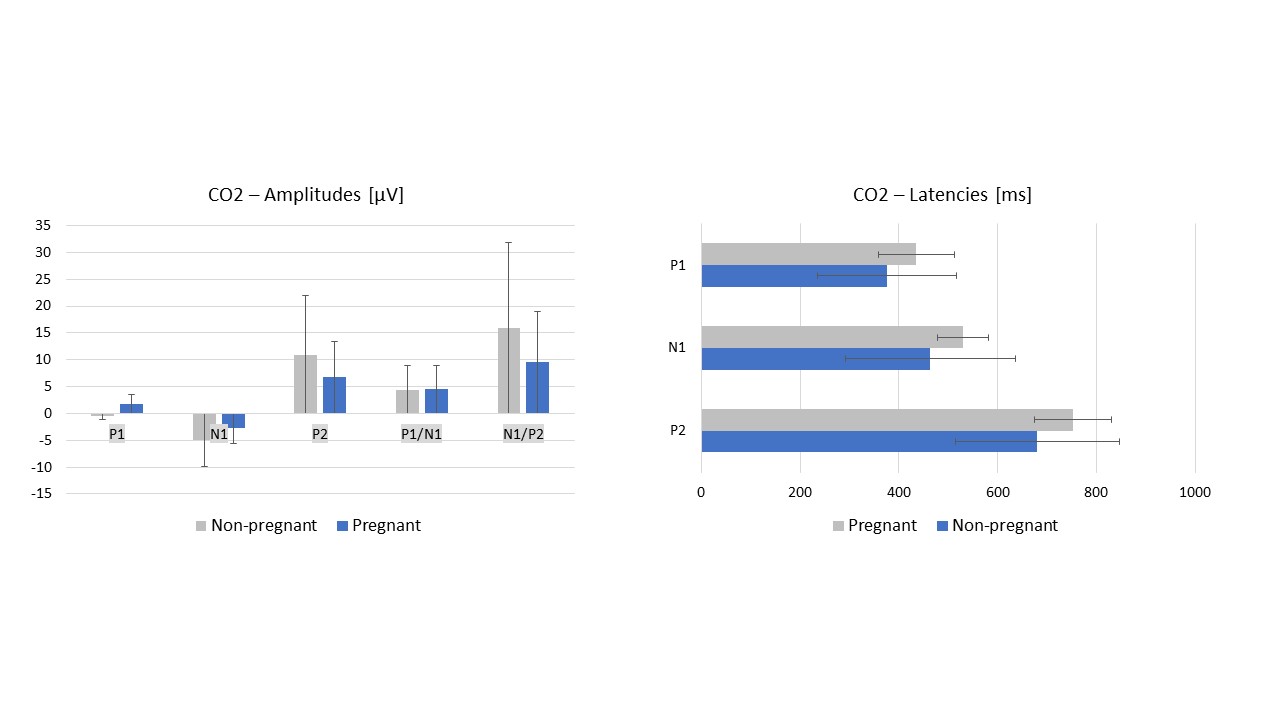

Supplement: Supplementary file 1 — Supporting Information [file BRB3-14-e3597-s001.docx]
